# Resting behaviour of *Aedes aegypti* in Trinidad: with evidence for the re-introduction of indoor residual spraying (IRS) for dengue control

**DOI:** 10.1186/1756-3305-6-255

**Published:** 2013-09-03

**Authors:** Dave D Chadee

**Affiliations:** 1 Department of Life Sciences, The University of the West Indies, St. Augustine, Trinidad, West Indies

**Keywords:** Trinidad, *Aedes aegypti*, Resting sites, Quiescence, Blood feeding, IRS, Dengue control

## Abstract

**Background:**

Historically, *Aedes aegypti* (L.) mosquitoes were controlled/eradicated by fumigation, residual spraying and the elimination of breeding sites. However, the underlying mechanisms of how these vector populations were managed have never been evaluated. Most studies report that these programs failed due to the emergence of DDT resistance in the 1950s and early 1960s. Therefore, behavioural and physiological factors have never been examined to determine program success or failure.

**Methods:**

A ten- week study collecting resting and flying mosquitoes from every room in houses using small hand nets and Propokock aspirators in St. Augustine, Trinidad, West Indies was conducted during the rainy season months of October to December 2010. During this period a laboratory study was also conducted to determine how soon after egg laying individual females took a blood-meal.

**Results:**

The field study showed the major resting sites of *Ae. aegypti* were bed rooms (81.9%), living rooms (8.7%) and kitchen (6.9%). The laboratory study showed only 10% of females accepted a blood meal immediately after oviposition but the majority, 70% accepted a blood meal 12 hours post oviposition.

**Conclusions:**

The results provide evidence for the efficacy of indoor residual spraying (IRS) and recommend its re-introduction by targeting the major resting sites of these mosquitoes, especially during dengue fever outbreaks.

## Background

Studies on the control of *Aedes aegypti* (L) the vector of urban yellow fever and dengue fever have yielded inconclusive results and therefore are not prescriptive [1]. The ‘dengue belt’ which runs between latitudes of 35°N and 35°S is home to over 3 billion people who are at risk of being infected. Each year, over 50 million cases of dengue fever, 600,000 cases of dengue haemorrhagic fever and its complications and 93 million asymptomatic cases are reported [2,3]. In the Americas, over 1.7 million cases of dengue fever have been reported with 50,000 clinically severe cases and 1,185 deaths reported in 2010, with an incidence of *circa* 200 cases per 100,000 population in many countries in Latin America and the Caribbean region [1,3].

Historically, control of *Ae. aegypti* was based on fumigation and reduction in the number of mosquito breeding sites [4]. With the introduction of DDT, both malaria and *Ae*. *aegypti* were controlled in some countries and eradicated in others [5]. However, the underlying mechanisms of how these vector populations were managed have never been scientifically evaluated [1]. Most studies report that these programs failed due to DDT resistance in the early 1960s but, few studies have examined why DDT was efficacious prior to developing resistance [1]. Even today many workers continue to advocate control methods for *Ae. aegypti* that include environmental sanitation and source reduction to destroy mosquito breeding sites and space spraying and ultra low volume spraying to kill flying adults [6,7]. However, Chadee [8] demonstrated the failure of the ULV application by truck- mounted machines, because the insecticides did not penetrate closed doors and windows and were blocked by barriers such as perimeter fences constructed of solid bricks.

The efficacy of focal treatment of breeding sites is well documented [5], but success of this strategy depends on numerous factors including; 1) goodwill of communities disposing them to allow access to their homes and to participate in environmental clean-up campaigns [1]; 2) political will to maintain continuous program funding [1,9]: 3) susceptibility of *Ae. aegypti* to the insecticides being used [10] and 4) adequate work ethic of vector control management and field staff [1].

Studies conducted in Africa [11], South East Asia [12] and Australia [13] have reported on the elusive resting habits of *Ae. aegypti*[14]. In the Americas, however, reports on the seclusive resting behaviour of *Ae. aegypti* within homes have come from mainly Panama [7], Costa Rica [15], Dominican Republic [16], Puerto Rico [17] and Mexico [18]*.* In addition, studies have shown dengue infected *Ae. aegypti* in and around houses in Asia [19] and in the Americas [20]. These studies suggest that houses provide suitable environment for man-vector contact, dengue transmission and suitable resting sites for adult mosquitoes.

Recently Chadee [1] reviewed emergency control measures for *Ae. aegypti* and reported the efficacy of indoor residual spraying (IRS) or intradomicillary spraying and insecticide treated curtains [18]. This strategy is based on the behaviour of *Ae. aegypti* which usually rests on wall surfaces before and after blood feeding, thus bringing the mosquito into contact with the insecticide treated material or wall surface resulting in high mortality rates. To date, three studies have provided indirect support for the re-introduction of IRS to control *Ae. aegypti* mosquitoes*,* with two studies providing evidence needed to support this recommendation. Chadee [21] evaluated focal treatment, ULV and IRS and reported effective control but was unable to explain the reasons for the significant reductions in the mosquito populations. The “cardinal points” study reported that 67% of the dengue positive houses harboured *Ae. aegypti* mosquitoes [22] while the “casa segura” concept reported the collection of dengue positive mosquitoes inside houses 27 days after initial clinical diagnosis [23]. Therefore the “cardinal points” [24] and the “casa segura” [25] studies provide further evidence of the presence of *Ae. aegypti* within homes. However, these studies did provide information on the mechanisms which control *Ae. aegypti* resting behaviour, information necessary for developing more scientifically sound evidence-based control strategies for both the present and the future.

The present study was conducted to determine the primary resting places of *Ae. aegypti* in houses in Curepe, Trinidad, West Indies and how soon after laying eggs females take a blood meal. These results reveal the behavioural and physiological processes which may account for the presence or resting behaviour of *Ae. aegypti* inside houses and have implications for dengue outbreak interventions.

## Methods

### Study site

This study was conducted for ten weeks at an urban housing centre located along the east-west-corridor [24] in north Trinidad: St. Augustine (10° 38’N; 60° 23’W) an urban university town with 3,000 houses and approximately 15,000 people. Trinidad traditionally experiences two annual seasons, the wet season occurring between the months of June to December and the dry season during the months of January to May. The average temperature ranges from 22°C to 30.5°C but is generally hotter in the wet season than in the dry season. This study was conducted during the rainy season, months of October to December 2010.

### Mosquito collections

Collections were made by two workers using small hand nets and using Propokack aspirators [25]. Collections were made from 0700 to 1500 hrs and included all rooms within the home. Indoor collections involved inspections of every room, and any resting mosquitoes were aspirated in a manner similar to that described by Garcia-Rejon et al. [23]. All adults were placed into labelled vials containing moistened cotton and taken to the Parasitology laboratory, Department of Life Sciences, University of the West Indies. Any flying mosquitoes observed were collected using hand nets. The collection of adults using the Propokack aspirator usually lasted about 15 minutes per home.

In the laboratory all mosquitoes collected were identified to species using a stereo-microscope and standard taxonomic keys [26]. Blood-feeding status of females was characterized using Christopher’s stages for gonotrophic development by placing one ovary on a glass slide to determine the ovary parity rates [27]. In addition, the blood-feeding status of female mosquitoes was determined by examining their abdomens while the Sella stages 1 to VII determined in accordance with the methodology described by Detinova [27].

### Laboratory studies

The *Ae. aegypti* strain used during these studies originated in St. Joseph, Trinidad, collected as eggs from June to August (1986) and designated the Trinidad strain. The mosquito strain used, their rearing conditions and methodology have been previously described [28]. Female adults were selected so that the post-emergence age of each was the same, and known to within 1 hour. On the third day post emergence, a sample of females was confirmed as inseminated by *post-mortem* dissection. Thereafter, females were allowed to engorge on blood from an experimenter’s arm within a 20 min period centred on 17.20 hr, a time close to the main peak of landing and biting of *Ae. aegypti* in the field in Trinidad [29].

### Experiments

On the fourth day post emergence, engorged females (assessed as such by eye) were placed individually, one per oviposition cage (30 × 30 × 30 cm) consisting of white cloth netting enclosing a wooden frame and containing a cube of white sugar in an uncovered Petri dish in the centre of the cage. In each cage, eight numbered small white polyethylene tub (SWT) (diameter of top 8.2 cm and bottom 7 cm, height 5.8 cm, capacity 300 ml) painted black outside, containing 200 ml of temperature-equilibrated tap water with the inside of each tub lined with a white paper towel were placed as described in the oviposition assay method developed by Corbet and Chadee [28].

In order to determine whether females rested or took a blood meal soon after oviposition, it was necessary to monitor when eggs were laid, that is to determine the diel oviposition periodicity. The oviposition periodicity was monitored by manually placing eight pre-prepared SWTs into each cage labeled in accordance with the cage number. The eight SWTs were exposed for intervals of 2 hours and removed and replaced with another set bearing the time of exposure and cage number. These females were monitored every 2 h for 48 hours. After each 2-h exposure sequence, all oviposition paper towels were removed from the SWTs and placed onto white sheets of paper towel on the laboratory bench and eggs counted under a stereomicroscope at × 40 magnification see [28]*.*

After oviposition had occurred one group of 50 females was individually offered a blood meal as described above between 18.00 h ± 20 min on post oviposition day 1 (POD) and again at 06.30 h ± 20 min the following morning. After this, blood meals were offered continuously at the above mentioned times until the 2^nd^ gonotrophic cycle was completed.

The number of blood feeding episodes recorded after oviposition was analyzed to determine the rest-time required for females to resume blood feeding and this data were analyzed by transforming the data into contingency tables and a G-test applied as well as a Chi square test [30].

### Ethical approval

This study was approved by the Law, Governance and Ethics Committee of the South West Regional Health Authority, Ministry of Health, San Fernando, Trinidad, West Indies.

## Results

### Adult collections

From 500 houses inspected, 31.5% (159) were positive for resting adult *Ae. aegypti* in St. Augustine, Trinidad. From a total of 2,039 resting *Ae. aegypti* mosquitoes collected, 57% were females (Table 1). Seventy-nine of the total mosquitoes captured while flying but were not included in the resting collections. Inspections of the St. Augustine study site found 9.2% of the houses infested with immature stages (Tables 1 and 2). Males were collected in 8.2% of the premises while females were collected in 22.9% of these premises. Bedrooms were the most favoured resting site of *Ae. aegypti,* followed by living rooms and kitchens (Table 2). More *Ae. aegypti* males were collected from the living room and kitchen than females (Table 2).

**Table 1:** **Adult *Aedes aegypti *collected from different rooms in houses from St. Augustine, Trinidad**

	**Females**				**Males**			
**Room**	**No. collected**	**%**	**Range**	**No. (%) of houses with females**	**No. collected**	**%**	**Range**	**No. (%) of houses with males**
Bedroom	968	81.9	0-32	159 (31.8)	493	57.4	0-29	130 (26.0)
Living room	101	8.7	0-10	93 (18.6)	201	23.4	0-25	71 (14.2)
Kitchen	71	6.0	0-6	40 (8.0)	93	10.8	0-22	32 (6.4)
Bathroom	22	1.8	0-5	40 (8.0)	55	6.4	0-19	29 (5.8)
Other rooms	19	1.6	0-12	12 (2.4)	17	2.0	0-13	10 (2.0)
Total	1181	100			858	100		

**Table 2:** **Periods of quiescence among *Aedes aegypti *females**

**Physiological process**	**Period of inactivity (h)**	**References**
Post adult emergence	24	Bowen, 1991
Post insemination	12	Fuchs & Kang, 1978
Post blood feeding	12	Klowden & Briegel, 1994
Inhibition of host seeking	24	Klowden, 1994
Post oviposition	12	Chadee, 2012

### Post-oviposition blood feeding

**Figure 1:**
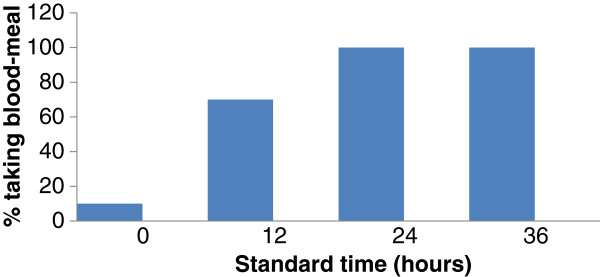
***Aedes aegypti*: times of accepting blood meals when offered immediately, 12 hours, 24 hours and 36 hours after oviposition.**

Five (10%) *Ae. aegypti* females accepted a blood meal immediately after oviposition (hour 0) while at post-oviposition hour 12, significantly (G = 510.87, P > 0.02) more females accepted a blood meal, that is 35 (70%) accepted a blood meal (Figure 1). At post-oviposition 36 hours, all females accepted a blood meal. Sixty-five percent of the females took two or more blood meals over the three successive days.

## Discussion

The results of the present study quantified the resting behaviour of *Ae. aegypti* in St. Augustine, Trinidad, W.I. with most females collected from bed rooms (81.9%), living rooms (8.7%) and kitchens (6.9%). Most resting mosquitoes were collected from dark wall surfaces close to the floor rather than on the upper surfaces of the walls close to the ceiling. These results are very similar to that observed in Panama [7], Costa Rica [15], Dominican Republic [16], Puerto Rico [17] and Mexico [18].

Numerous physiological factors may be responsible for the resting behaviour observed during this study. Chadee and Beier [31] reported that *Ae. aegypti* mosquitoes processed blood by pre-diuresis during blood feeding and by diuresis on completion of blood feeding, especially while resting on clothing or under furniture and on the dark lower part of the wall surface close to the floor. This resting activity is confirmed by studies conducted by Klowden and Briegel [32] who suggested that after a critical threshold in blood-feeding is reached, females usually rest for two physiological processes to occur: first for blood-induced-distension of the gut wall which triggers a refractory period during which females no longer seek or take blood meals [33]; and second, if the blood meal initiates vitellogenesis, a second inhibition accompanies oocyte development [34,35] and this haemo-lymphborne factor is produced circa 30 hours post blood feeding. This inhibition normally lasts until oviposition and is often referred as the oocyte-induced-inhibition [35]. Based on a preliminary laboratory study females were often observed to remain stationary and seldom move from their resting place until pre-oviposition flight (Chadee, Unpub data). Studies conducted by Bowen [36], Fuch and Kang [37], Klowden and Briegel [32], Klowden [33] and Chadee [38] suggest that *Ae. aegypti* females required periods of “quiescence” or resting periods (Table 2). These studies clearly demonstrated that females rest for between 36-50 hours post blood-feeding. In fact, *Ae. aegypti* females collected while resting indoors in Panama were parous (48.5%) and possibly contained oocytes of various Christophers stages and can be considered old females.

During the present study only 10% of the females offered a blood-meal immediately after oviposition, fed but the majority required a rest of at least 12 hours before taking a blood-meal (Figure 1) a similar percentage to that reported by Chadee [38]. Results from these various studies suggest that *Ae. aegypti* females remain indoors or at resting sites for long periods and this may explain the failure of conventional ultra-low-volume (ULV) spraying using either aerial or truck mounted applications to kill or reduce vector populations. Studies conducted in Panama explained that the resting behaviour of *Ae. aegypti* was largely responsible for the failure of the spray programs because little or no insecticide aerosol droplets reached these indoor areas especially in bed rooms [7,16]. The location of these resting sites was also a primary reason for the failure to control *Ae. aegypti* in Trinidad [8], Suriname [6], Dominican Republic [16], Panama [17] and Jamaica [39] since physical barriers such as block fences and closed doors and windows impeded the penetration or movement of the insecticides from the roadside into houses [8,38].

**Figure 2:**
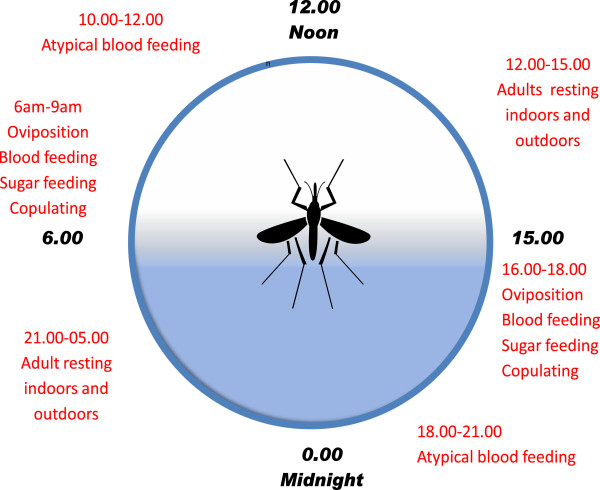
***Aedes aegypti *circadian rhythms observed in Trinidad.**

Conversely, these results suggest that insecticide applications targeting the resting sites of *Ae. aegypti* mosquitoes may be more effective in reducing or controlling these vector populations. Therefore, the re-introduction of intra-domiciliary spraying or indoor residual spraying (IRS) may prove to be a viable option based on the evidence provided in this study. That is controlling dengue using an old method but with new understanding of the underlying behavioural mechanisms of the mosquito. For example, the success of the early malaria programs in the 1950s and 1960s using IRS for the application of DDT resulted in the successful control/eradication of *Ae. aegypti* mosquitoes in some countries in the Americas [5]. The initial success of this program was based on the resting behaviour of *Ae. aegypti* on wall surfaces before and after blood feeding which brings the mosquito into contact with the insecticide treated material or wall surface while the mosquitoes are undergoing diuresis, a physiological process which removes excess liquid from the blood meal and results in compact red blood cells which can be used for egg production. During this post-feeding period females fly to suitable resting sites or remain at the site if not disturbed for the completion of vitellogenesis after which pre-oviposition flight occurs to suitable breeding sites. In addition, this resting notion is supported by studies on *Ae. aegypti* circadian rhythms in Trinidad which demonstrated that peak blood feeding, oviposition, sugar feeding and copulation occurred between 06-09 hours and at 16-18 hours (Figure 2), with most females resting for circa 19 hours except for some atypical biting activity reported by Chadee and Martinez [29].

The present study therefore now provides the evidence for the efficacy of IRS programs and explains why some countries in the past were successful in controlling and/or eradicating malaria and *Ae. aegypti* mosquitoes [5]. Therefore, the challenge for the present and future will be to re-introduce IRS into the dengue control armoury especially as part of an integrated vector management program (IVM). IRS would be suitable for both emergency and routine control programs but IVM managers should ensure the prudent use of insecticides by using 3 options currently available rather than repeatedly applying the same insecticide each year as outlined by World Health Organization [40]. Firstly, by alternating the use of different insecticides in time and space which reduces the frequency of resistance, secondly, by using a combination of different insecticide classes and thirdly using mixtures of two insecticides for coordinated/synergistic action which will further delay the onset of resistance [40]. The use of both old and new insecticides which have been found to have long residual activity would be ideal (e.g. paint-based mixtures of insecticides or entomopathogenic fungi) thus delaying the development of insecticide resistance. In addition, IVM managers should elicit political support for the retraining of staff using the IRS techniques and to expand space spraying (fogging) programs which, under suitable circumstances, can also penetrate inside houses and kill resting mosquitoes.

## Conclusions

This study provides the evidence for the efficacy of IRS in controlling the *Ae. aegypti* mosquitoes and dengue fever transmission by highlighting the major behavioural and physiological factors which contribute to their resting behaviour inside houses in St. Augustine, Trinidad.

## Abbreviations

IRS: Indoor residual spraying; IGR: Insect growth regulator; DDT: Dichlorodiphenyltrichloroethane; ULV: Ultra-low-volume.

## Competing interests

The author declares that he has no competing interests.
